# Effects of statin use on volumetric mammographic density: results from the KARMA study

**DOI:** 10.1186/s12885-015-1457-9

**Published:** 2015-05-28

**Authors:** Ida Skarping, Judith S. Brand, Per Hall, Signe Borgquist

**Affiliations:** 1 Division of Oncology and Pathology, Department of Clinical Sciences, Lund University, Skåne University Hospital, SE-221 85 Lund, Sweden; 2 Department of Medical Epidemiology and Biostatistics, Karolinska Institute, Stockholm, Sweden; 3 Department of Oncology, Skåne University Hospital, Lund, Sweden

**Keywords:** Volumetric mammographic density, Statins, Screening-based cohort, Epidemiology, Breast cancer

## Abstract

**Background:**

Epidemiological data on statins and breast cancer risk have been inconclusive. The aim of this study was to clarify the role of statins in breast cancer risk by studying their effect on mammographic density.

**Methods:**

The KARolinska MAmmography project for risk prediction of breast cancer (KARMA) includes 70,877 women who underwent either a screening or clinical mammography from January 2011 to December 2013. In total, 41,102 women responded to a web-based questionnaire, and had raw digital mammograms stored. Volumetric mammographic density was measured using Volpara™ and information on statin use was obtained through linkage with the Swedish National Prescription Register. Analysis of covariance was used to study the effect of statin use on mammographic density, adjusting for a large set of potential confounders. We also studied the effects of statin class and treatment duration and tested for potential effect modification by hormone replacement therapy (HRT).

**Results:**

Statin use was recorded in 3,337 women (8.1 %) of the study population and lipophilic statins was the most commonly prescribed type (93.4 % of all statin users). After multivariable adjustment, percent dense volume was lower in statin users than in non-users (P < 0.001). This association was explained by a larger absolute non-dense volume in statin users (P < 0.001). Overall, no difference in absolute dense volume was detected, but interaction analyses revealed a larger dense volume among statin users who reported ever HRT use (P = 0.03). No differential effects were observed according to statin lipophilicity and treatment duration.

**Conclusions:**

We observed no overall effect of statin use on mammographic density in terms of absolute dense volume, although a larger absolute dense volume was observed in statin users who reported ever HRT use, which requires further investigation.

## Background

Statins are inhibitors of 3-hydroxy-3-methyl-glutaryl co-enzyme A (HMG-CoA) reductase and are used worldwide as an efficient cholesterol-lowering medication with proven anti-inflammatory properties [1]. By reducing HMG-CoA reductase activity, levels of mevalonate decrease, which influences important down-stream players for cellular pathways that are crucial for cancer initiation, growth, and metastasis [2]. Pre-clinical studies and phase II trials have demonstrated anti-carcinogenic properties of statins [3–5]. Large-scale epidemiological observational studies have reported conflicting findings regarding the role of statins in breast cancer prevention [6–11]. On the other hand, available data on breast cancer prognosis are more consistent showing a lower risk of recurrence in lipophilic statin users [12–14]. These data suggest that statins may not prevent cancer occurrence, e.g. by affecting the malignant genotype, but rather inhibit the progress of an existing cancer by affecting the malignant phenotype [11]. Mammographic density (MD), which reflects the amount of fibroglandular or radio dense tissue on an X-ray of the breast (mammogram), is a strong determinant of breast cancer risk [15, 16]. Women with extremely dense breasts (>75 % dense tissue on a mammogram) have a 4–6 fold higher risk of developing breast cancer compared to women having fatty or non-dense breasts (<5 % dense tissue) [15]. MD also shares many risk factors with breast cancer and is therefore seen as an intermediate in breast cancer etiology [17]. Several determinants of high MD have also been proven to cause cancer [18] and MD may be a useful marker in studies aiming at elucidating the role of potential risk factors for breast cancer [19, 20]. To clarify the effect of statins on breast cancer risk, we decided to study the association between statin use and volumetric mammographic density in a large-screening based cohort with comprehensive information on medication use, lifestyle factors and volumetric mammographic density.

## Methods

### Study population

The KARolinska MAmmography project for the risk prediction of breast cancer (KARMA) is a prospective cohort study initiated in January 2011 and comprises 70,877 women who attended mammography screening or clinical mammography at four hospitals in Sweden [21]. Participants responded to a detailed web-based questionnaire, and permission was asked for storage of “for processing” (raw) full-field digital mammograms (FFDM) and linkage to Swedish national registers on inpatient care, prescriptions, cancer, and cause of death.

For the present study, we selected all women who attended the mammography screening program (40–74 years) with raw digital mammograms stored at baseline (N = 50,461). We excluded women with previous cancers other than non-melanoma skin cancer (N = 3,015), women who underwent breast enlargements/reductions/surgery (N = 2,191), women lacking information on age and body mass index (BMI) (N = 3,908), and women pregnant 12 months prior to study entry (N = 40), leaving 41,307 women in the study. Of these, 41,102 women had medio-lateral oblique (MLO) mammograms and represented the final study population. The study was approved by the ethical review committee at the Karolinska Institute (Stockholm, Sweden), and all participants provided written informed consent.

### Statin exposure

Information on statin use was obtained through linkage to the Swedish Prescribed Drug Register [22], which has had nationwide coverage since mid-2005. The register covers all drugs sold and dispensed by prescription at Swedish pharmacies to patients with a personal identification number. The following Anatomical Therapeutic Chemical (ATC) codes were extracted: ‘C10AA01’ (simvastatin), ‘C10AA04’ (fluvastatin), ‘C10AA05’ (atorvastatin) and ‘C10AA08’ for lipophilic statins, and ‘C10AA03’ (pravastatin) and ‘C10AA07’ (rosuvastain) for hydrophilic statins. Women were considered to be current users if they were dispensed statins during the year before mammography. We also calculated and categorized the cumulative statin duration in current users in three-year categories (<2 years, 2–5 years, >5 years).

### Measurement of mammographic density

Mammographic density was measured from baseline mammograms using a fully automated volumetric method, Volpara, which has been validated against breast magnetic resonance imaging (MRI) data and appears to be robust to changes in imaging conditions [23]. We have previously shown that Volpara performs well in a high-throughput setting with both percent and absolute dense volumes being associated with established density determinants and breast cancer risk [24]. For our analyses, we used the average mammographic density from the left and right breast of the MLO view.

Technical details of the Volpara algorithm have been described elsewhere [23]. In brief, the algorithm computes the thickness of dense tissue at each pixel using the X-ray attenuation of an entirely fatty region as an internal reference. The absolute dense volume (cm^3^) is measured by integrating the dense thickness at each pixel over the whole mammogram, and the total breast volume (cm^3^) is derived by multiplying the breast area by the recorded breast thickness with an appropriate correction for the breast edge. The percent dense volume (%) is obtained from the ratio of these two measures, and the absolute non-dense volume is derived by subtracting the absolute dense volume from the total breast volume.

### Covariate assessment

Information on potential confounders was derived from the web-based questionnaire. The questionnaire covers a wide range of breast cancer risk factors and other baseline characteristics such as anthropometrics, alcohol and smoking, education, reproductive and hormonal factors (age at menarche, number of births and age at first birth, menstruation status, use of oral contraceptives and hormone replacement therapy (HRT)), and family history of breast cancer. Body mass index (BMI) was calculated based on self-reported weight and height. Menopausal status was defined according to information on menstruation status, previous oophorectomy, and age at study entry. Women with no periods during the last year, a history of oophorectomy, or age 55 or older were defined as postmenopausal. Information on other medications was extracted from the Swedish Prescribed Drug Register using the following ATC codes: ‘B01AC06’ (low-dose aspirin) and ‘A10BA02’ (metformin). Current use was defined by dispensing within the year preceding mammography.

### Statistical methods

Characteristics of statin users were compared with those of non-users using Chi-square tests for categorical variables and T-tests for continuous variables. All mammographic measures were log-transformed to approximate the normal distribution. We used analysis of covariance (ANCOVA) to examine associations between statin use and mammographic density, and geometric means were calculated for each category of statin use. We also studied the effect of statin class (lipophilic vs. hydrophilic) and treatment duration. Women using both lipophilic and hydrophilic statins were included in analyses of overall statin use and treatment duration but were excluded from analysis by statin class (N = 46).

All analyses were adjusted for potential confounders in a stepwise manner. We started with an age-adjusted model, after which BMI was added. In the third model, we further adjusted the analyses for other known determinants of mammographic density confounders, including menopausal status, HRT use, parity/age at first birth, age at menarche, education level, smoking, alcohol consumption, and history of benign breast disease. In the final multivariable adjusted model, we added co-medication including low-dose aspirin and metformin use.

Since the effect of statins on breast cancer risk may depend on HRT use [25], we also tested for potential effect modification by HRT by adding a multiplicative interaction term between statin and any history of HRT use to the multivariable adjusted models. All statistical analyses were conducted using STATA version 12.0 (Stata Corp. College Station, TX, USA). Seventeen percent of the women had missing values on one or more covariates. On the basis of the missing data pattern, we assumed the missing values to be “missing at random” [26], and imputed these values using a multiple imputation technique (10 imputation sets) [27].

## Results

Table 1 shows the characteristics of statin users and non-users. Statin use was recorded in 3,337 women (8.1 %) of the KARMA study population. As expected, statin users were more likely to be of older age, to be postmenopausal, and to have a higher BMI and a smoking history. Statin users also more often reported HRT use in the past and were more frequently on low-dose aspirin and metformin medication. Other variables associated with statin use were age at menarche, age at first birth, oral contraceptive use, and alcohol intake.

**Table 1:** Descriptive characteristics of the study population, overall and stratified by current statin use

Participant characteristic^a^	Total	Statin use		P value
		No	Yes	
		N = 37,765	N = 3,337	
Age (years), mean (SD)	55.0 (9.8)	54.2 (9.6)	63.8 (6.9)	<0.001
Body mass index (kg/m^2^), mean (SD)	25.4 (4.2)	25.2 (4.2)	27.1 (4.6)	<0.001
Age at menarche (years), mean (SD)	13.1 (1.5)	13.1 (1.5)	13.3 (1.5)	<0.001
Parity, % (N)				0.54
0	12.1 (4,979)	12.2 (4,582)	11.9 (397)	
1	14.1 (5,792)	14.0 (5,294)	15.0 (498)	
2	48.5 (19,901)	48.5 (18,296)	48.2 (1,605)	
> = 3	25.3 (10,363)	25.3 (9,533)	24.9 (830)	
Age at first birth (years), mean (SD)	27.1 (5.2)	27.2 (5.2)	25.0 (4.6)	<0.001
Menopausal status, % (N)				<0.001
Premenopausal	40.2 (16,506)	43.1 (16,272)	7.0 (234)	
Perimenopausal/unknown	3.3 (1,349)	3.5 (1,304)	1.4 (45)	
Postmenopausal	56.6 (23,247)	53.5 (20,189)	91.6 (3,058)	
Oral contraceptive use, % (N)	79.7 (32,602)	80.5 (30,257)	70.9 (2,345)	<0.001
Hormone replacement therapy, % (N)				<0.001
Never	80.5 (30,791)	81.7 (28,809)	65.6 (1,982)	
Former	15.5 (5,945)	14.3 (5,040)	30.0 (905)	
Current	4.0 (1,521)	4.0 (1,395)	4.2 (126)	
Benign breast disease, % (N)	22.1 (8,919)	22.0 (8,156)	23.3 (763)	0.09
Family history of breast cancer, % (N)	13.2 (5,236)	13.2 (4,815)	13.2 (421)	0.91
Low-dose aspirin use, % (N)	4.7 (1,911)	2.2 (833)	32.3 (1,078)	<0.001
Metformin use, % (N)	1.6 (666)	0.7 (263)	12.1 (403)	<0.001
Education, % (N)				<0.001
Compulsory	14.4 (5,705)	12.9 (4,726)	31.2 (979)	
Gymnasium	31.6 (12,557)	31.9 (11,664)	28.5 (893)	
University	54.0 (21,440)	55.2 (20,178)	40.3 (1,262)	
Smoking status, % (N)				<0.001
Never	48.2 (19,750)	48.8 (18,400)	40.6 (1,350)	
Former	39.7 (16,269)	39.1 (14,726)	46.4 (1,543)	
Current	12.2 (4,984)	12.1 (4,551)	13.0 (433)	
Alcohol (g/week), % (N)				<0.001
0	19.4 (7,912)	18.9 (7,090)	24.9 (822)	
1–25	24.0 (9,788)	24.3 (9,110)	20.5 (678)	
25–50	28.1 (11,471)	28.4 (10,640)	25.2 (831)	
50–100	14.6 (5,949)	14.6 (5,481)	14.2 (468)	
>100	13.9 (5,666)	13.8 (5,162)	15.3 (504)	

^a^Missingness on all variables < 5 %, except for age at first birth (5.6 %) and hormone replacement therapy (6.9 %)

The distribution of statin use by lipophilicity, type, and duration is summarized in Table 2. Most statin users were prescribed a lipophilic statin (93.4 %), with simvastatin being the most commonly prescribed type. Among current users, the percentages of participants using statins <2 years, 2–5 years, and >5 years were 15.1 %, 38.5 % and 46.4 % respectively.

**Table 2:** Details of statin use in current users (N = 3,337)

	% (N)
Number of statins used	
1	96.1 (3,208)
2	3.8 (126)
3	0.1 (3)
Statin class^a^	
lipophilic	93.4 (3,074)
hydrophilic	6.6 (217)
Statin type^a^	
simvastatin	81.9 (2,627)
atorvastatin	11.3 (363)
fluvastatin	0.1 (4)
rosuvastatin	4.4 (140)
pravastatin	2.3 (74)
Statin duration	
<2 years	15.1 (503)
2–5 years	38.5 (1,286)
>5 years	46.4 (1,548)

^a^Exclusive use only. Lipophilic statins: simvastatin, atorvastatin, fluvastatin, pitavastatin; Hydrophilic statins: rosuvastatin, pravastatin

Table 3 shows the geometric means of mammographic density by statin use, lipophilicity, and statin duration. Overall, percent dense volume was lower in statin users than in non-users (*P* < 0.001). The largest difference in percent dense volume was observed in the age-adjusted model, while the effect of statin use was attenuated after adjustment for BMI, resulting in smaller difference in geometric means despite being significant. Inspection of the individual density components revealed that the inverse association with percent dense volume was attributed to a larger absolute non-dense volume in statin users (*P* < 0.001). No effect of statin use on the absolute dense volume was found after multivariable adjustment, although a trend towards a higher dense volume was observed after adjusting for concomitant use of low-dose aspirin and metformin (*P* = 0.06). There was also no evidence of differential effects by lipophilicity and treatment duration according to overlapping confidence intervals. Both lipophilic and hydrophilic statin users had a lower percent dense volume and higher absolute non-dense volume compared to non-users. The differences in density measures by statin duration was driven by the strong overall effect of statin use, without a clear trend by length of exposure in current users as demonstrated by the U-shaped associations.

**Table 3:** Geometric means (95 % CI) of volumetric mammographic density measures by current statin use

		Geometric mean (95 % CI)^a^			
		Model 1	Model 2	Model 3	Model 4
	Statin use				
Percent dense volume (%)	No	8.01 (7.97–8.05)	7.93 (7.90–7.96)	7.93 (7.90–7.96)	7.94 (7.91–7.97)
	Yes	6.89 (6.77–7.01)	7.73 (7.63–7.84)	7.74 (7.63–7.85)	7.66 (7.54–7.77)
	*P* value	<0.001	0.001	0.001	<0.001
Absolute dense volume (cm^3^)	No	56.8 (56.6–57.1)	57.1 (56.8–57.3)	57.1 (56.8–57.3)	57.0 (56.8–57.3)
	Yes	60.7 (59.7–58.5)	57.6 (56.7–58.5)	57.5 (56.7–58.4)	58.0 (57.0–59.0)
	*P* value	<0.001	0.31	0.32	0.06
Absolute non-dense volume (cm^3^)	No	643 (639–647)	653 (650–656)	653 (650–656)	652 (649–655)
	Yes	808 (791–826)	676 (666–686)	675 (665–685)	689 (678–700)
	*P* value	<0.001	<0.001	<0.001	<0.001
	Statin type				
Percent dense volume (%)	None	8.01 (7.98–8.05)	7.93 (7.90–7.97)	7.93 (7.90–7.97)	7.94 (7.91–7.97)
	Lipophilic	6.92 (6.80–7.04)	7.73 (7.65–7.87)	7.77 (7.66–7.88)	7.69 (7.57–7.81)
	Hydrophilic	6.46 (6.05–6.89)	7.32 (6.94–7.71)	7.34 (6.97–7.73)	7.26 (6.90–7.65)
	*P* value	<0.001	<0.001	<0.001	<0.001
Absolute dense volume (cm^3^)	None	56.8 (56.6–57.1)	57.1 (56.8–57.3)	57.1 (56.8–57.3)	57.0 (56.8–57.3)
	Lipophilic	60.8 (59.8–61.8)	57.7 (56.8–58.6)	57.7 (56.8–58.6)	58.1 (57.2–59.1)
	Hydrophilic	58.2 (54.7–61.8)	55.0 (51.9–58.3)	54.8 (51.8–58.1)	55.3 (52.2–58.6)
	*P* value	<0.001	0.20	0.66	0.21
Absolute non-dense volume (cm^3^)	None	643 (639–647)	653 (650–656)	653 (650–656)	652 (649–655)
	Lipophilic	807 (789–825)	675 (664–685)	674 (664–685)	688 (676–699)
	Hydrophilic	830 (765–900)	683 (646–723)	679 (642–718)	693 (655–733)
	*P* value	<0.001	<0.001	0.03	0.003
	Statin duration				
Percent dense volume (%)	None	8.01 (7.97–8.05)	7.93 (7.90–7.96)	7.93 (7.90–7.96)	7.94 (7.91–7.97)
	<2 years	7.00 (6.71–7.31)	7.54 (7.29–7.81)	7.64 (7.38–7.90)	7.57 (7.32–7.84)
	2–5 years	6.76 (6.58–6.95)	7.59 (7.43–7.76)	7.63 (7.47–7.80)	7.56 (7.40–7.73)
	>5 years	6.96 (6.79–7.14)	7.92 (7.77–8.08)	7.86 (7.71–8.02)	7.77 (7.61–7.96)
	*P* trend	<0.001	0.03	0.01	<0.001
Absolute dense volume (cm^3^)	None	56.8 (56.6–57.1)	57.1 (56.8–57.3)	57.1 (56.8–57.3)	57.0 (56.8–57.3)
	<2 years	58.5 (56.2–60.9)	56.6 (54.5–58.8)	57.0 (54.9–59.2)	57.3 (55.2–59.5)
	2–5 years	60.6 (59.1–62.2)	57.5 (56.2–58.9)	57.8 (56.4–59.2)	58.2 (56.8–59.7)
	>5 years	61.4 (60.0–62.9)	57.9 (56.6–59.2)	57.5 (56.2–58.8)	58.0 (56.7–59.4)
	*P* trend	<0.001	0.20	0.32	0.06
Absolute non-dense volume (cm^3^)	None	643 (639–647)	653 (650–656)	653 (650–656)	652 (649–655)
	<2 years	767 (727–809)	683 (659–709)	680 (655–705)	689 (664–715)
	2–5 years	825 (798–854)	690 (674–706)	689 (673–705)	702 (685–719)
	>5 years	809 (784–834)	661 (647–676)	663 (649–677)	678 (662–693)
	*P* trend	<0.001	0.001	<0.001	<0.001

^a^All volumetric mammographic density measures were log transformed for analysis and the values shown are on a back-transformed scale

*P* value refers to F-test for between-statin group differences in geometric means, while *P* trend refers to linear trend analyses entering statin duration (<2 years, 2–5years, > 5 years) as continuous term in the analysis

Model 1: adjusted for age (years)

Model 2: Model 1 + body mass index (kg/m^2^)

Model 3: Model 2 + menopausal status (pre, peri/unknown, post), HRT use (never, former, current), parity/age at first birth (nulliparous, 1 child age at first birth < 25 years, 1 child age at first birth ≥ 25 years, 2 children age at first birth < 25 years , 2 children age at first birth ≥ 25 years, ≥ 3 children age at first birth < 25 years, ≥ 3 children age at first birth ≥ 25 years), age at menarche (years), education level (compulsory, gymnasium, university), smoking (never, former, current), alcohol consumption (nondrinker, 0,1–1,9 g/day, 2.0–4.9 g/day, 5.0–9.9 g/day, ≥10 g/day) and benign breast disease (yes vs. no) +

Model 4: Model 3 plus low-dose aspirin (yes vs. no) and metformin use (yes vs. no)

Interaction analyses showed evidence of effect modification by HRT. A positive association between statin use and the absolute dense volume was found in those who had used HRT, while no such association was found in never HRT users (*P*_interaction_ = 0.03) (Table 4).

**Table 4:** Multivariable adjusted geometric means (95 % CI) of volumetric mammographic density measures

			Geometric mean (95 % CI)^a^		
	N	Statin use	Percent dense volume (%)	Absolute dense volume (cm3)	Absolute non-dense volume (cm3)
BMI < 25 kg/m2	22,385	No	10.1 (10.0–10.1)	51.6 (51.3–52.0)	455 (452–457)
		Yes	9.5 (9.3–9.7)	53.0 (51.5–54.5)	498 (485–511)
		P value	<0.001	0.09	<0.001
BMI 25–30 kg/m2	13,273	No	6.5 (6.5–6.6)	62.5 (62.0–62.9)	887 (882–893)
		Yes	6.4 (6.2–6.5)	62.5 (61.1–64.0)	914 (895–933)
		P value	0.04	0.94	0.01
BMI > 30 kg/m2	5,444	No	4.8 (4.7–4.8)	68.9 (68.1–69.6)	1362 (1350–1416)
		Yes	4.8 (4.7–4.9)	69.9 (67.9–72.0)	1385 (1352–1419)
		P value	0.88	0.38	0.23
Premenopausal	17,855	No	9.7 (9.7–9.8)	62.9 (62.4–63.3)	573 (569–576)
		Yes	9.3 (8.8–9.7)	62.1 (58.6–65.9)	598 (567–632)
		P value	0.04	0.69	0.12
Postmenopausal	23,247	No	6.8 (6.8–6.8)	52.9 (52.6–53.2)	720 (716–724)
		Yes	6.5 (6.4–6.6)	53.9 (53.1–54.8)	767 (755–779)
		P value	<0.001	0.04	<0.001
Never HRT^b^	14,407	No	6.7 (6.7–6.8)	52.9 (52.5–53.2)	724 (719–729)
		Yes	6.4 (6.3–6.5)	53.4 (52.3–54.5)	760 (747–774)
		P value	<0.001	0.38	<0.001
Ever HRT^b^	6,475	No	6.9 (6.8–6.9)	53.0 (52.5–53.6)	723 (718–729)
		Yes	6.6 (6.5–6.8)	55.2 (53.7–56.7)	771 (755–787)
		P value	0.02	0.01	<0.001
P interaction^b^			0.61	0.03	0.11

^a^All mammographic density measures were log transformed prior to analysis and the values shown are on a back-transformed scale. ^b^In postmenopausal women only. *P* value refers to F-test for between-statin group differences in geometric means. *P* interaction refers to the *P* value of the multiplicative interaction term

Model 4: Adjusted for age (years), body mass index (kg/m^2^), menopausal status (pre, peri/unknown, post), HRT use (never, former, current), parity/age at first birth (nulliparous, 1 child age at first birth < 25 years, 1 child age at first birth ≥ 25 years, 2 children age at first birth < 25 years, 2 children age at first birth ≥ 25 years, ≥ 3 children age at first birth < 25 years, ≥ 3 children age at first birth ≥ 25 years), age at menarche (years), education level (compulsory, gymnasium, university), smoking (never, former, current), alcohol consumption (nondrinker, 0,1–1,9 g/day, 2.0–4.9 g/day, 5.0–9.9 g/day, ≥10 g/day) and benign breast disease (yes vs. no), low-dose aspirin use (yes vs. no) and metformin use (yes vs. no)

## Discussion

In this study including 41,102 women from a large screening-based cohort, we found no evidence of an overall effect of statin use on absolute dense volume. Statin users had a significantly lower percent dense volume, but this difference was mainly attributable to a larger non-dense volume in statin users. While a large number of studies have investigated the effect of statin use on breast cancer risk and prognosis [6–14], only few studies have examined the association with mammographic density. Our results are in line with a longitudinal study showing no effect of statins on change in area-based mammographic density [28]. No differential effects were observed by statin class or treatment duration [13, 29] but interaction analyses revealed potential effect modification by HRT with a larger absolute dense volume among statin users who also reported HRT use.

Mammographic density can be expressed in both absolute and relative terms. The preferred MD measure for breast cancer risk prediction is debated, although recent studies show that the proportion of dense breast tissue may have a stronger predictive value than the absolute amount of dense tissue, thus suggesting non-dense tissue as a contributing risk factor [30, 31]. Percent dense volume mirrors the relationship between dense and non-dense parenchyma of the breast. Because the proportion of dense volume is strongly influenced by the amount of non-dense tissue in the breast, any factor that is associated with a difference in non-dense volume can lead to a substantial difference in percent dense volume, even if there is no association with the absolute dense volume. Since most statin users have a medical history of hypercholesterolemia and are more likely to have larger fat deposits than non-users, their lower percent dense volume, as observed in this study, is most likely due to the underlying indication, as reflected by their larger absolute non-dense volume. We therefore believe that the absolute dense volume is a more optimal measure for studying the effect of statins on mammographic density, as this measure is less influenced by this type of confounding [32].

The role of statins in primary breast cancer prevention is controversial, and several factors need to be acknowledged when interpreting the available evidence. First of all, our study highlights the issue of confounding by indication, an effect that cannot easily be controlled for in observational studies [33]. Any protective effect of statins may be counteracted in observational analyses due to the underlying indication of dyslipidemia, as high total cholesterol and low HDL cholesterol levels have been associated with an increase in breast cancer risk in postmenopausal women [34, 35], although a recent publication report opposite findings with positive associations between HDL and breast cancer risk [36]. The notion that statin use and body composition are strongly interrelated, another effect not easily adjusted for in association analyses, may also partly explain the conflicting observational data regarding the effect of statins on breast cancer risk [6–8, 13].

Another shortcoming in many studies may be the lack of separate analyses for lipophilic and hydrophilic statins [7]. Theoretically, lipophilic statins, in contrast to hydrophilic statins, are able to penetrate the cell membrane and affect cell proliferation and survival, hence having anti-carcinogenic effects [37]. Regarding uptake in the hepatocyte cell, lipophilic statins are passively diffused through membranes, whereas hydrophilic statins have an active carrier-mediated uptake [38]. The different mechanisms of uptake generate less hepato-selectivity in lipophilic statins [38]. Animal studies have shown greater distribution in extrahepatic tissue for lipophilic statins [39]. Thus, lipophilic statins, in comparison to hydrophilic statins, have superior influence on extrahepatic tissue. However, in this study, analyses stratified for statin lipophilicity did not show a significant difference in absolute dense volume. Finally, bioavailability differs among statins, and the distribution to different tissues is dose-dependent [2]. The effectiveness of statins, which were initially designed as hepatoselective drugs [40], has been questioned as a cancer-preventive drug considering its low systemic availability [10]. Recently, we conducted a window-of-opportunity trial, prescribing a high-dose lipophilic statin (atorvastatin) to newly diagnosed breast cancer patients two weeks prior to primary breast cancer surgery. The study showed significantly reduced proliferation (Ki67) in the subgroup of patients where tumors expressed the target for statins, HMG-CoA reductase [4]. These results imply that statins may exert systemic effects including tumor-targeted effects in breast cancer, despite its hepatoselective properties. Similar anti-proliferative effect of lipophilic statins have been demonstrated in another window trial [5]. Therefore, statins and their role as anti-cancerous drug, requires further investigation, in particular long-term use of high-dose lipophilic statins [41].

Despite the lack of an overall association with statin use, the higher absolute dense volume found in statin users who also reported HRT is of interest. HRT use is associated with an increase in MD [42] and breast cancer risk [43, 44], and the effect of HRT seems to depend on the pretreatment level of endogenous estrogens. Data from the Women’s Health Initiative trial show that the adverse effect of HRT on breast cancer risk is largest in those having the lowest estrogen levels [45]. Data on the effect of statins on endogenous estrogen levels are scarce, but a small biomarker study reported lower estrone sulfate levels in women using simvastatin [28]. Given the larger HRT effect in a low endogenous estrogen environment, this may explain the larger absolute dense volume observed with statins in ever HRT users. Moreover, an interaction between statin and HRT use has previously been reported for breast cancer [25], in a direction similar to that observed for the absolute dense volume. An alternative explanation for the observed interaction is the underlying indication. A metabolite of cholesterol has been associated with estrogen positive breast cancers [46], supporting a cross talk between estrogen and cholesterol in breast cancer. Thus, the larger absolute dense volume in statin users who ever used HRT could also reflect the combined effect of HRT and underlying disease of hypercholesterolemia (rather than the actual statin prescription) on mammographic density. Since mammographic density represents one of the strongest risk factors for breast cancer, potential adverse effects of statins and/or hypercholesterolemia in women using HRT require further exploration.

Our study has several strengths, including the large screening-based cohort with detailed information on lifestyle factors as well as registry-based information on statin use and other medications. We used a fully-automated method for measuring mammographic density, which in contrast to other methods is not prone to subjective measurement error. Moreover, volumetric approaches yield more accurate density measures, as they account for inter-individual differences in breast thickness. Both dense and non-dense tissues were evaluated in this study, which strengthens the interpretation of our findings and highlighted the issue of confounding by indication. Our study also has some limitations. The cross-sectional nature limits the ability to explore the temporal association between statins and mammographic density in terms of initiation and discontinuation. Also, we could not study the effect of long-term treatment (>10 years), because the population-based prescription register has only reached full coverage since 2005. Finally, we did not have data available on the dosages prescribed.

## Conclusions

In conclusion, we found no strong evidence for an overall effect of statin use on volumetric mammographic density in terms of absolute dense volume. The observed interaction with HRT with a larger absolute dense volume in statin users, who also reported HRT use, requires further investigation.

## Abbreviations

MD: mammographic density
KARMA: KARolinska MAmmography project for the risk prediction of breast cancer
HRT: Hormone replacement therapy
HMG-CoA: 3-hydroxy-3-methyl-glutaryl co-enzyme A
FFDM: Full-field digital mammograms
BMI: Body mass index
MLO: Medio-lateral oblique
ATC: Anatomical Therapeutic Chemical
MRI: Magnetic resonance imaging

## References

[1] Lefer DJ (2002). Statins as potent antiinflammatory drugs. Circulation.

[2] Chan KK, Oza AM, Siu LL (2003). The statins as anticancer agents. Clinical cancer research.

[3] Muck AO, Seeger H, Wallwiener D (2004). Inhibitory effect of statins on the proliferation of human breast cancer cells. International journal of clinical pharmacology and therapeutics.

[4] Bjarnadottir O, Romero Q, Bendahl PO, Jirstrom K, Ryden L, Loman N (2013). Targeting HMG-CoA reductase with statins in a window-of-opportunity breast cancer trial. Breast cancer research and treatment.

[5] Garwood ER, Kumar AS, Baehner FL, Moore DH, Au A, Hylton N (2010). Fluvastatin reduces proliferation and increases apoptosis in women with high grade breast cancer. Breast cancer research and treatment.

[6] Cauley JA, Zmuda JM, Lui LY, Hillier TA, Ness RB, Stone KL (2003). Lipid-lowering drug use and breast cancer in older women: a prospective study. Journal of Women's Health (1540–9996).

[7] Bonovas S, Filioussi K, Tsavaris N, Sitaras NM (2005). Use of statins and breast cancer: a meta-analysis of seven randomized clinical trials and nine observational studies. Journal of Clinical Oncology: official journal of the American Society of Clinical Oncology.

[8] Cauley JA, McTiernan A, Rodabough RJ, LaCroix A, Bauer DC, Margolis KL (2006). Statin use and breast cancer: prospective results from the Women's Health Initiative. Journal of the National Cancer Institute: JNCI.

[9] McDougall JA, Malone KE, Daling JR, Cushing-Haugen KL, Porter PL, Li CI (2013). Long-term statin use and risk of ductal and lobular breast cancer among women 55 to 74 years of age. Cancer epidemiology, biomarkers & prevention: a publication of the American Association for Cancer Research, cosponsored by the American Society of Preventive Oncology.

[10] Undela K, Srikanth V, Bansal D (2012). Statin use and risk of breast cancer: a meta-analysis of observational studies. Breast cancer research and treatment.

[11] Graaf MR, Beiderbeck AB, Egberts AC, Richel DJ, Guchelaar HJ (2004). The risk of cancer in users of statins. Journal of Clinical Oncology: official journal of the American Society of Clinical Oncology.

[12] Nielsen SF, Nordestgaard BG, Bojesen SE (2012). Statin use and reduced cancer-related mortality. The New England journal of medicine.

[13] Ahern TP, Pedersen L, Tarp M, Cronin-Fenton DP, Garne JP, Silliman RA (2011). Statin prescriptions and breast cancer recurrence risk: a Danish nationwide prospective cohort study. Journal of the National Cancer Institute: JNCI.

[14] Murtola TJ, Visvanathan K, Artama M, Vainio H, Pukkala E (2014). Statin use and breast cancer survival: a nationwide cohort study from Finland. PloS one.

[15] McCormack VA, dos Santos SI (2006). Breast density and parenchymal patterns as markers of breast cancer risk: a meta-analysis. Cancer Epidemiology, Biomarkers & Prevention: A Publication of the American Association for Cancer Research.

[16] Yaghjyan L, Colditz GA, Collins LC, Schnitt SJ, Rosner B, Vachon C (2011). Mammographic breast density and subsequent risk of breast cancer in postmenopausal women according to tumor characteristics. Journal of the National Cancer Institute.

[17] Boyd NF, Rommens JM, Vogt K, Lee V, Hopper JL, Yaffe MJ (2005). Mammographic breast density as an intermediate phenotype for breast cancer. The lancet oncology.

[18] Boyd NF, Lockwood GA, Martin LJ, Knight JA, Byng JW, Yaffe MJ (1998). Mammographic densities and breast cancer risk. Breast disease.

[19] Carney PA, Miglioretti DL, Yankaskas BC, Kerlikowske K, Rosenberg R, Rutter CM (2003). Individual and combined effects of age, breast density, and hormone replacement therapy use on the accuracy of screening mammography. Annals of internal medicine.

[20] Mandelson MT, Oestreicher N, Porter PL, White D, Finder CA, Taplin SH (2000). Breast density as a predictor of mammographic detection: comparison of interval- and screen-detected cancers. Journal of the National Cancer Institute.

[21] The KARMA study http://karmastudy.org/sources/

[22] Wettermark B, Hammar N, Fored CM, Leimanis A, Otterblad Olausson P, Bergman U (2007). The new Swedish Prescribed Drug Register–opportunities for pharmacoepidemiological research and experience from the first six months. Pharmacoepidemiology and drug safety.

[23] Harvey RHSMBMJYNKJ: Robust Breast Composition Measurement - Volpara™. *Digital Mammography* 2010(10th International Workshop, IWDM 2010, Girona, Catalonia, Spain, June 16–18, 2010. Proceedings):pp 342–349.

[24] Brand JS, Czene K, Shepherd JA, Leifland K, Heddson B, Sundbom A (2014). Automated measurement of volumetric mammographic density: a tool for widespread breast cancer risk assessment. Cancer epidemiology, biomarkers & prevention: a publication of the American Association for Cancer Research, cosponsored by the American Society of Preventive Oncology.

[25] Beck P, Wysowski DK, Downey W, Butler-Jones D (2003). Statin use and the risk of breast cancer. Journal of clinical epidemiology.

[26] Sterne JA, White IR, Carlin JB, Spratt M, Royston P, Kenward MG (2009). Multiple imputation for missing data in epidemiological and clinical research: potential and pitfalls. Bmj.

[27] Schafer JL (1997). Analysis of incomplete multivariate data.

[28] Higgins MJ, Prowell TM, Blackford AL, Byrne C, Khouri NF, Slater SA (2012). A short-term biomarker modulation study of simvastatin in women at increased risk of a new breast cancer. Breast cancer research and treatment.

[29] Ahern TP, Lash TL, Damkier P, Christiansen PM, Cronin-Fenton DP (2014). Statins and breast cancer prognosis: evidence and opportunities. The lancet oncology.

[30] Byrne C, Schairer C, Wolfe J, Parekh N, Salane M, Brinton LA (1995). Mammographic features and breast cancer risk: effects with time, age, and menopause status. Journal of the National Cancer Institute: JNCI.

[31] Pettersson A, Graff RE, Ursin G, Santos Silva ID, McCormack V, Baglietto L, Vachon C, Bakker MF, Giles GG, Chia KS *et al.*: Mammographic Density Phenotypes and Risk of Breast Cancer: A Meta-analysis. *Journal of the National Cancer Institute* 2014, 106(5). 10.1093/jnci/dju078 PMC4568991 24816206

[32] Schetter SE, Hartman TJ, Liao J, Richie JP, Prokopczyk B, DuBrock C, Signori C, Hamilton C, Demers LM, El-Bayoumy K *et al.*: Differential impact of body mass index on absolute and percent breast density: implications regarding their use as breast cancer risk biomarkers. *Breast cancer research and treatment* 2014. 10.1007/s10549-014-3031-6 24951269

[33] Bosco JL, Silliman RA, Thwin SS, Geiger AM, Buist DS, Prout MN (2010). A most stubborn bias: no adjustment method fully resolves confounding by indication in observational studies. Journal of clinical epidemiology.

[34] Kitahara CM, Berrington de Gonzalez A, Freedman ND, Huxley R, Mok Y, Jee SH (2011). Total cholesterol and cancer risk in a large prospective study in Korea. Journal of clinical oncology : official journal of the American Society of Clinical Oncology.

[35] Furberg AS, Veierod MB, Wilsgaard T, Bernstein L, Thune I (2004). Serum high-density lipoprotein cholesterol, metabolic profile, and breast cancer risk. Journal of the National Cancer Institute.

[36] Martin LJ, Melnichouk O, Huszti E, Connelly PW, Greenberg CV, Minkin S, Boyd NF: Serum lipids, lipoproteins, and risk of breast cancer: a nested case–control study using multiple time points. *Journal of the National Cancer Institute* 2015, 107(5). 10.1093/jnci/djv032 PMC4822522 25817193

[37] Demierre MF, Higgins PD, Gruber SB, Hawk E, Lippman SM (2005). Statins and cancer prevention. Nature Reviews Cancer.

[38] Schachter M (2005). Chemical, pharmacokinetic and pharmacodynamic properties of statins: an update. Fundamental & Clinical Pharmacology.

[39] Nezasa K, Higaki K, Matsumura T, Inazawa K, Hasegawa H, Nakano M (2002). Liver-specific distribution of rosuvastatin in rats: comparison with pravastatin and simvastatin. Drug metabolism and disposition : the biological fate of chemicals.

[40] Hamelin BA, Turgeon J (1998). Hydrophilicity/lipophilicity: relevance for the pharmacology and clinical effects of HMG-CoA reductase inhibitors. Trends in pharmacological sciences.

[41] Berstein LM (2005). Clinical usage of hypolipidemic and antidiabetic drugs in the prevention and treatment of cancer. Cancer Letters.

[42] Greendale GA, Reboussin BA, Slone S, Wasilauskas C, Pike MC, Ursin G (2003). Postmenopausal hormone therapy and change in mammographic density. Journal of the National Cancer Institute: JNCI.

[43] Fournier A, Fabre A, Mesrine S, Boutron-Ruault MC, Berrino F, Clavel-Chapelon F (2008). Use of different postmenopausal hormone therapies and risk of histology- and hormone receptor-defined invasive breast cancer. Journal of clinical oncology: official journal of the American Society of Clinical Oncology.

[44] Borgquist S, Anagnostaki L, Jirstrom K, Landberg G, Manjer J (2007). Breast tumours following combined hormone replacement therapy express favourable prognostic factors. International journal of cancer Journal international du cancer.

[45] Farhat GN, Parimi N, Chlebowski RT, Manson JE, Anderson G, Huang AJ (2013). Sex hormone levels and risk of breast cancer with estrogen plus progestin. Journal of the National Cancer Institute.

[46] Nelson ER, Wardell SE, Jasper JS, Park S, Suchindran S, Howe MK (2013). 27-Hydroxycholesterol links hypercholesterolemia and breast cancer pathophysiology. Science.

